# The role of hypertension and diabetes mellitus in the severity of gait and balance disturbances in iNPH

**DOI:** 10.1186/2045-8118-12-S1-O11

**Published:** 2015-09-18

**Authors:** Simon Agerskov, Maria Wallin, Per Hellström, Carsten Wikkelsö, Mats Tullberg

**Affiliations:** 1 Hydrocephalus research unit at the institute of neuroscience and physiology, the Sahlgrenska Academy at the University of Gothenburg, Sweden

## Background

Idiopathic normal pressure hydrocephalus (iNPH) is a disorder characterized by gait and balance disturbances, urinary incontinence and cognitive impairment. Of these three, gait and balance disorders are often the first to manifest and also the group that improve the most after shunt surgery. Vascular risk factors (particularly hypertension and diabetes mellitus) has been shown to increase the risk of developing iNPH. There is however further need to investigate if these risk factors have any impact on the severity of symptoms and improvement after shunt surgery.

The aim of this study is to examine the connection between hypertension, diabetes mellitus (DM) and the severity of gait and balance disturbances in patients diagnosed with iNPH pre- and postoperativley. The aim was also to determine if patients with risk factors present show less improvement in gait and balance after surgery compared to controls.

## Methods

A local database containing clinical data on all iNPH-patients diagnosed and treated in Gothenburg between 1978 and 2013 was used. The database contains a total of 389 patients diagnosed in accordance with the American-European guidelines. Gait and balance were measured using: a seven step scale (higher score indicating a more severe disturbance), the Romberg test, TUG-test and the 10m walking test. All tests were performed and recorded by physiotherapists with a special interest in iNPH. Hypertension and diabetes was defined in accordance with the ICD coding system. Differences between groups were tested using the Mann-Whitney U test.

## Results

Patients who had a documented history of hypertension scored significantly higher on the gait scale (mean score: 3.14 vs 2.65, p<0.001) and also performed worse on all the measured tests. Patients with DM also scored higher on the grading scale (mean: 3.26 vs 2,78, p=0.006) and performed worse on the majority of tests compared to controls. After shunt surgery, patients with hypertension or DM still performed at a significantly lower levels compared to controls but did not show a worse relative improvement in gait and balance on the grading scale nor on other tests.

The results are preliminary and will be expanded.

## Conclusions

The presence of hypertension and/or diabetes mellitus not only seems to increase the risk of developing iNPH but seem to accelerate the progression of symptoms. Patients who have either hypertension or DM perform significantly worse in test measuring gait and balance compared to controls before and after shunt surgery. The existence of cardiovascular risk factors does not however, seem to impact the postoperative relative improvement in gait and balance after shunt surgery.

## References

[B1] Jaraj D Agerskov S Rabiei K Marlow T Jensen C Guo X Kern S Wikkelsö C Skoog I Vascular Factors in Normal Pressure Hydrocephalus, submitted for review 10.1212/WNL.0000000000002369 PMC4762415 26773072

